# Atraumatic intra‐articular, extra‐synovial ganglion cyst of the lateral knee deep to the iliotibial band: A case report and review of the literature

**DOI:** 10.1002/ccr3.8501

**Published:** 2024-02-19

**Authors:** Alvarho Guzman, Nicholas Williams, Therese Dela Rueda, Edward Xiao, Arina Caliman, James L. Chen, Patrick J. McGahan

**Affiliations:** ^1^ Advanced Orthopaedics and Sports Medicine San Francisco California USA; ^2^ Albany Medical College Albany New York USA; ^3^ University of Connecticut School of Medicine Farmington Connecticut USA

**Keywords:** anatomy, orthopedics, sports medicine

## Abstract

**Key Clinical Message:**

We highlight the rare case of an atraumatic, intra‐articular ganglion cyst of the lateral knee deep to the iliotibial band that was successfully treated nonoperatively, a pathology yet to be reported in orthopedic literature.

**Abstract:**

Ganglion cysts are mucin‐filled synovial cysts commonly found on the dorsal surface of the hands and feet. Intra‐articular ganglion cysts of the knee are rare, and when they present clinically, are typically treated operatively through arthroscopic surgery. We present the first reported case of an atraumatic intraarticular, extra‐synovial ganglion cyst of the lateral knee located deep to the iliotibial band that was successfully treated without operative intervention through repeated intra‐articular aspirations of the knee.

## Graphical abstract

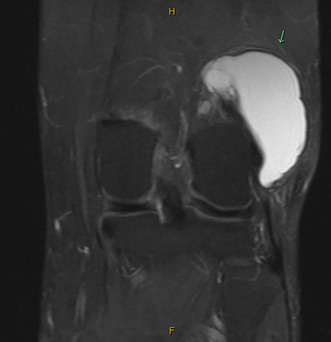

## 1 INTRODUCTION

Ganglion cysts (GC) are synovial cysts filled with gelatinous mucinoid material believed to arise from repetitive microtrauma resulting in mucinous degeneration of connective tissue.^1^ GC are commonly encountered in orthopedic practice and well known to occur in the dorsal wrist, palm, and shoulder, with up to 60% of GC occurring on the dorsal wrist.^2^ Furthermore, GC may arise from intra or extra‐articular, soft tissue, intraosseous, or periosteal locations with symptoms varying according to size and origin.^2^ Intra‐articular GC of the knee are rare and have been reported to arise from the cruciate ligaments, menisci, popliteal tendons, alar folds, and subchondral bone.^3^, ^4^, ^5^ Additionally, GC arising from the lateral portion of the knee have been described in recent literature following arthroscopic medial meniscus repair that were both subsequently excised surgically.^6^ This case report presents a case of an atraumatic intraarticular, extrasynovial ganglion cyst of the lateral knee located deep to the iliotibial (IT) band and superficial to both the fibular collateral ligament and lateral femoral condyle that was successfully treated nonoperatively through repeat joint aspirations. To our knowledge, this is the first reported case of this pathology that was treated without surgical intervention.

## 2 CASE REPORT

**FIGURE 1:**
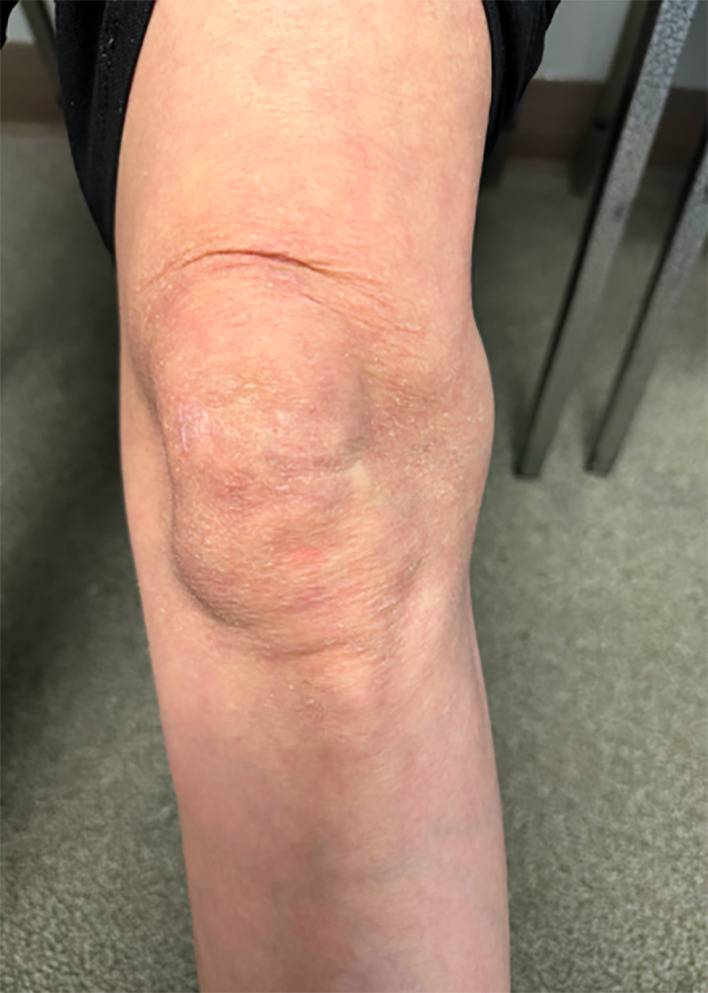
Physical examination of the left knee depicting 4 × 4 cm area of swelling over the lateral knee joint just posterior to the IT band and anterior to the biceps femoris.

A 61‐year‐old female presented to our orthopedic clinic with atraumatic left knee pain and swelling for the past 6 months. She reported lateral left knee pain, stiffness, and the inability to fully flex and extend knee. Additionally, she noted a soft‐tissue mass on her lateral left knee along the joint line that was exacerbated by walking and physical activity. Mild pain of the medial left knee was also reported by the patient. Six months prior to her visit to our clinic, she received plain film radiographs of the left knee that were normal, and an ultrasound of the left knee revealing a potential Baker's cyst. On physical examination of the left knee, the patient had full range of motion, mild patellofemoral crepitus, mild joint line tenderness of the medial knee, and ligamentous stability. Further inspection of the lateral left knee revealed a 4 × 4 cm area of swelling over the lateral joint just posterior to the IT band and anterior to the biceps femoris (Figure 1). The etiology of the cyst is atraumatic and unknown. No popliteal fossa swelling was present of the left knee and the patient was able to perform a single leg squat.

**FIGURE 2:**
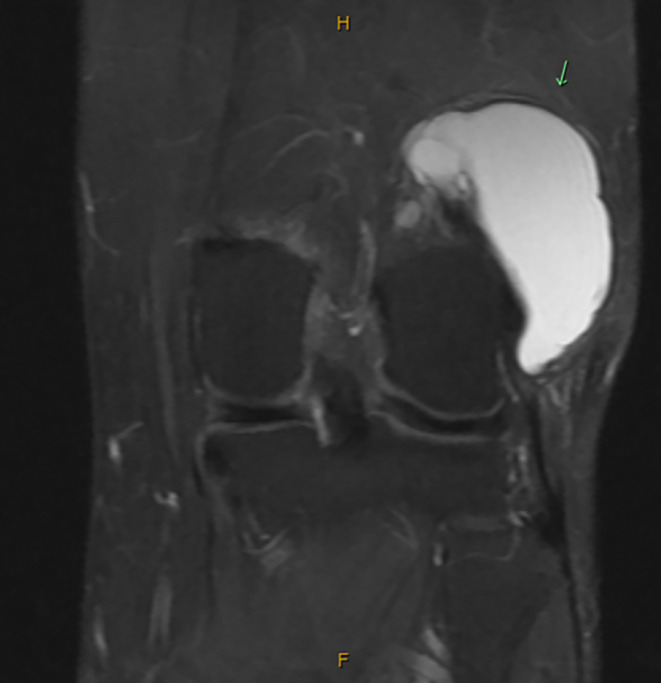
T2 coronal view MRI without contrast of the left knee depicting multiloculated cystic lesion deep to the IT band.

**FIGURE 3:**
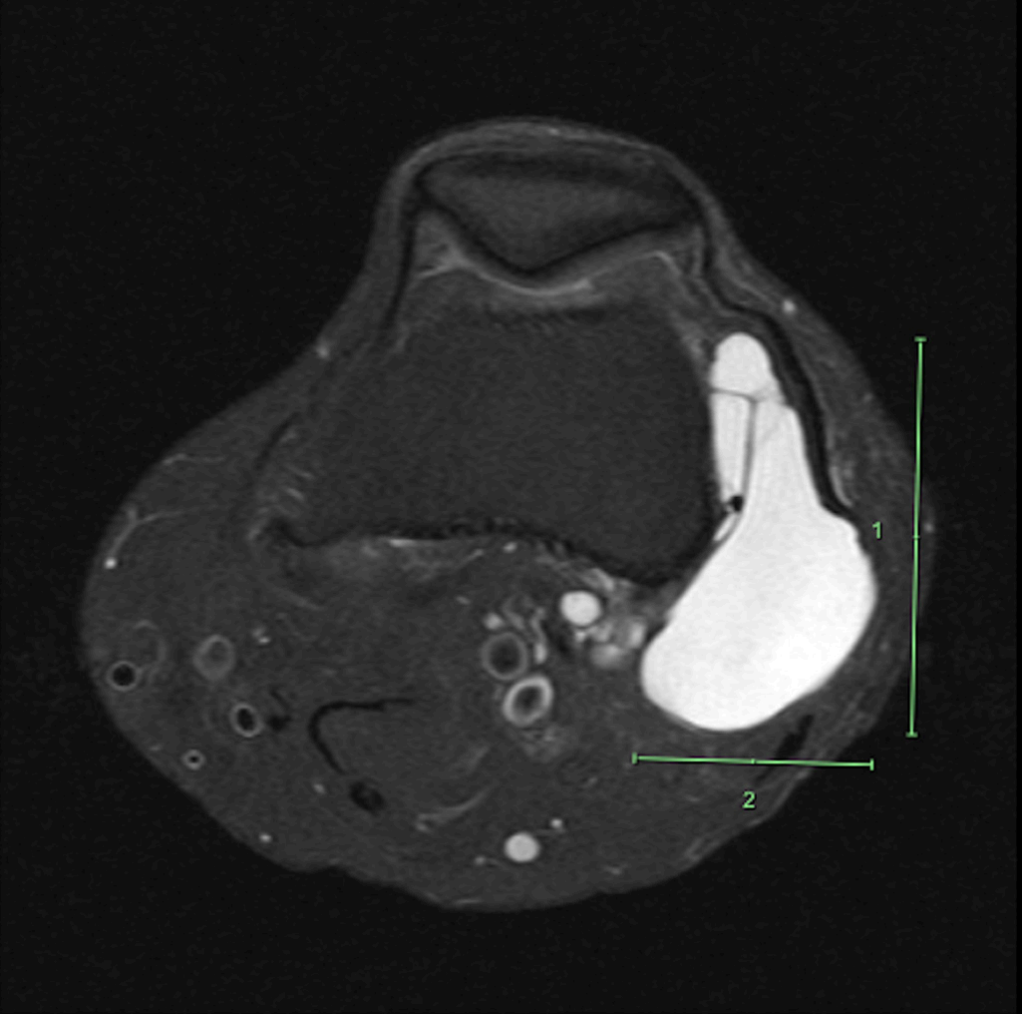
T2 axial view MRI without contrast of the left knee depicting multiloculated cystic lesion deep to the IT band.

**FIGURE 4:**
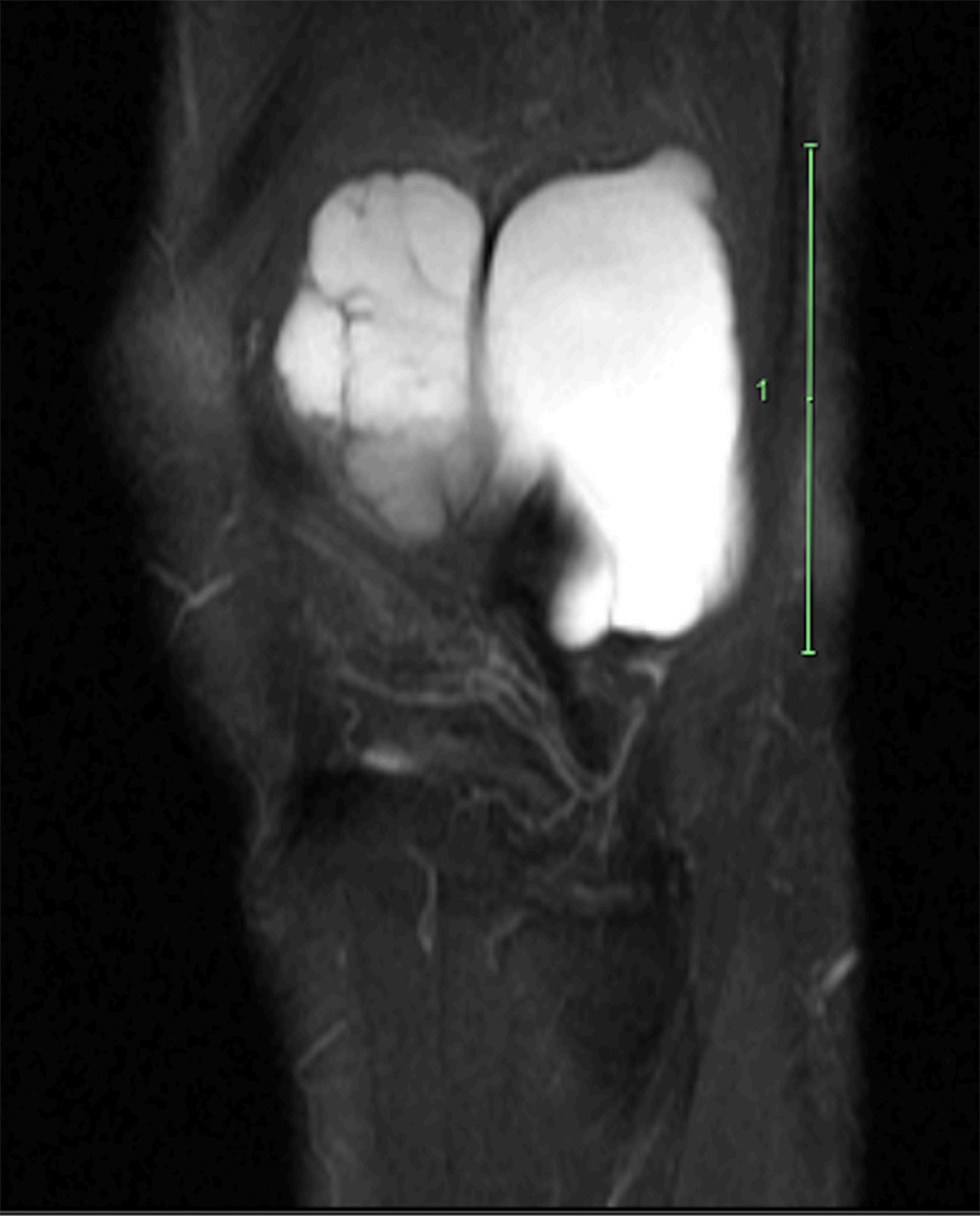
T2 sagittal view MRI without contrast of the left knee depicting multiloculated cystic lesion deep to the IT band.

Based on the patient history, clinical presentation, and physical examination findings, magnetic resonance imaging (MRI) of the left knee without contrast was ordered to further evaluate the soft‐tissue mass present over the lateral knee. Upon follow‐up presentation after MRI imaging was ordered, the patient reported minimal change in symptoms. MRI imaging of the left knee revealed a multilobulated, multiloculated cystic lesion located deep to the IT band, superficial to the fibular collateral ligament and lateral femoral condyle that extended posterior to the distal femoral diaphysis and measured approximately 5.8 cm CC by 5.4 cm anteroposterior (AP) by 3.3 cm mediolateral (ML) (Figures 2, 3, 4). It was also explained to the patient that the previous ultrasound report of the Baker's cyst in her left knee does not correlate to the location of her ganglion cyst, as the ganglion cyst is not within the popliteal fossa and is anatomically distinct.

**FIGURE 5:**
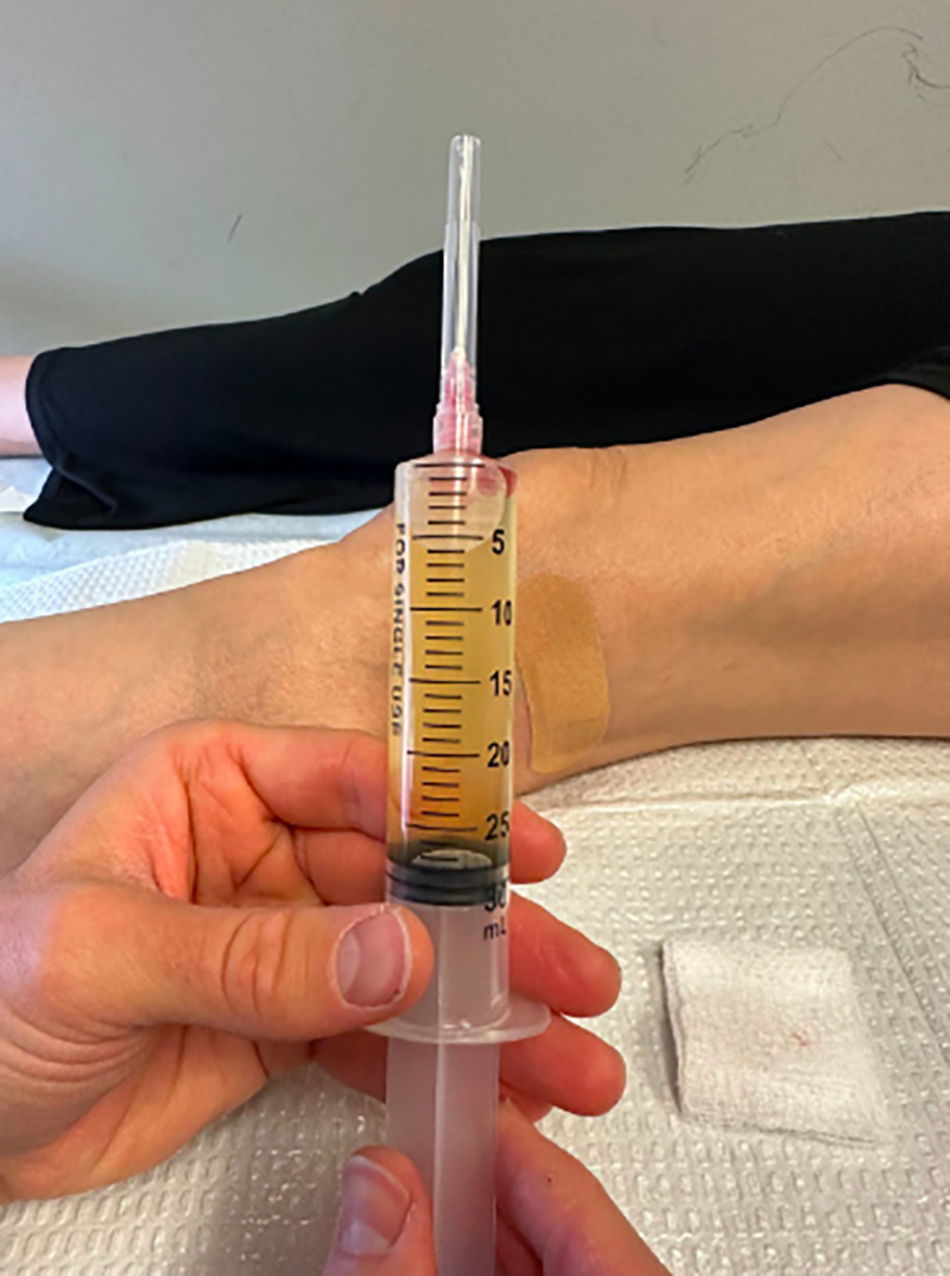
Depiction of 25 cc of yellow, gelatinous fluid aspirate from the knee.

Following discussion of imaging results, treatment options were discussed and included conservative management with rest, activity modification, and NSAIDs. Additionally, intra‐articular aspiration of the knee joint was offered to reduce synovial inflammation associated with the ganglion cyst. Surgical excision was not indicated nor recommended given the patient lacked neurovascular manifestations or severe, functionally limiting symptoms. The option of a corticosteroid injection was discussed, but the patient deferred and the decision to go forward without an injection was agreed upon. The patient elected to proceed with intra‐articular aspiration of the left knee, and 25 cc of yellow, gelatinous fluid was aspirated (Figure 5; Video S1). The need for further aspiration if swelling of the left knee persists was discussed with the patient and she agreed to follow‐up as needed depending on her symptoms.

The patient returned four times over the next 6 months due to symptomatic inflammation of the left knee and received aspiration three out of these four visits. In her second aspiration of the left knee, 1 month following her first aspiration, 15 cc of red gelatinous fluid was aspirated. One month later, the patient returned to the clinic with mild swelling of the left knee and it was explained to the patient that not enough fluid was present in the knee joint to prompt aspiration at this visit. One month later, the patient returned with increased pain and swelling of the left knee and 20 cc of yellow, gelatinous fluid was aspirated from the knee joint.

Following aspiration of the left knee, the patient was counseled to return to our clinic for repeat aspiration of the knee joint if the swelling continues to persist. She continues to be seen on a symptomatic basis and has not been seen for a repeat aspiration since her most recent aspiration over 2 months ago. The patient has not required additional injections beyond the three previously mentioned aspirations and has been able to avoid surgery with near elimination of her pain and complete restoration of knee mobility.

## 3 DISCUSSION

Intra‐articular GC of the knee are clinically rare in orthopedic practice but have been reported to occur in the cruciate ligaments, menisci, subchondral bone, alar folds, patellar tendon, and fat pad.^3^, ^4^, ^5^, ^6^, ^7^ Commonly detected as incidental findings on MRI, intra‐articular GC are reported to have a prevalence between 0.9% and 1.3%.^8^, ^9^ GC of the lateral knee has been described in the available literature, with two cases occurring following arthroscopic meniscectomy and an atraumatic case arising from Hoffa's fat pad, all of which were subsequently treated with surgical excision of the cyst with variable postoperative outcomes and no recurrence.^6^, ^7^ Furthermore, intraneural GC of the knee has been reported in patients presenting with foot drop and peripheral neuropathy, which were all from articular origin within the knee joint and subsequently excised surgically.^10^, ^11^ In contrast to prior reported cases of intra‐articular GC of the knee, our case report highlights the presentation of an intra‐articular, extra‐synovial ganglion cyst of the lateral knee that was successfully treated with repeat joint aspirations.

The exact pathogenesis of ganglion cyst formation within the joint remains controversial, although theories including herniation of synovial tissue through adjacent structures, displacement of synovial tissue during embryogenesis, and mucoid degeneration due to microtrauma have been proposed.^8^, ^12^ Clinical presentations of GC of the knee are variable, but can present with vague pain, swelling, stiffness, and limitation in range of motion as noted by our case presentation. Moreover, clinical presentation is dependent on the size and location of the cyst within the knee. Larger cysts and cysts near the femoral attachment of the anterior cruciate ligament are more likely to be symptomatic. Limitations in knee extension may occur when a GC is located anteriorly, or limitations in knee flexion if a GC is located posteriorly.^13^ MRI is the imaging assessment of choice used for the evaluation of GC of the knee since diagnosis cannot be based solely on clinical presentation due to variable presentation of symptoms and mass location.^14^

The proposed management for intra‐articular GC of the knee include arthroscopic excision, open excision, and nonoperative therapy including joint aspirations.^15^ According to current literature, arthroscopic excision is the most commonly used operative procedure for intra‐articular GC. The advantages include a low recurrence rate and the ability to take a biopsy specimen during arthroscopy and address any concomitant articular damage within the knee joint.^15^ Krudwig et al. analyzed 85 intra‐articular GC found during 8000 procedures managed subsequently with arthroscopic excision and noted acceptable clinical outcomes with no recurrence.^3^ Additionally, Brown and Dandy identified 38 cases of intra‐articular GC of the knee out of 6500 knee arthroscopies also subsequently treated with arthroscopic excision and reported good to excellent clinical outcomes in 95% of these patients.^16^ Open excision is used for large, extra‐articular GC of the knee and may be indicated in infrapatellar fat pad GC >4.5 cm in size.^12^, ^15^ In regard to nonoperative therapy, several studies have suggested no treatment for asymptomatic cysts found incidentally during MRI and have even noted shrinkage of symptomatic cysts with nonoperative therapy, although it is recommended to clinically monitor cysts in the event of future enlargement or compressive effects that may require surgery.^3^, ^17^, ^18^

In contrast to most reported literature on intra‐articular GC of the knee, we report the case of a patient who was successfully treated with repeat joint aspirations of the knee, rather than through arthroscopic excision. Intra‐articular GC aspiration of the knee is classically performed through ultrasonography or computed tomography (CT) guided aspiration and is associated with a higher possible recurrence than surgery.^19^, ^20^, ^21^ Antonacci et al. reported immediate pain relief in patients using CT‐guided aspiration in three patients with an ACL cyst, however, symptoms recurred in one patient after only 3 months.^22^ Demey et al. classified intra‐articular GC into liquid cysts and infiltrative cysts and compared outcomes of aspiration compared to arthroscopic excision, ultimately reporting superior outcomes in arthroscopic excision compared to aspiration.^23^ Additionally, this study reported patients with liquid cysts responded better to aspiration in comparison to infiltrative cysts.^23^ Despite the possibility of recurrence, aspiration of the knee can be performed in an outpatient setting and recovery is generally fast, making aspiration an attractive option for the active patient who wishes to abstain from surgery. Unlike the literature described, our patient received joint aspiration of the affected knee without ultrasonography and CT guidance and was not of highly active nature seeking a fast recovery. They continue to be seen on a symptomatic basis and have noted progressive relief with each subsequent aspiration.

## 4 CONCLUSION

To our knowledge, this is the first case highlighting a patient presenting with an atraumatic ganglion cyst of the lateral knee located deep to the IT that was treated nonoperatively through repeat joint aspirations. Our patient has not required additional aspirations beyond those mentioned in this case report, and has been able to avoid operative intervention with near elimination of pain and complete restoration of knee mobility. With this case report, we hope to add to existing orthopedic literature the successful nonoperative treatment of an atraumatic ganglion cyst of the lateral knee located deep to the IT band, a pathology that typically occurs following surgical intervention and is commonly treated arthroscopically.

## AUTHOR CONTRIBUTIONS

**Alvarho Guzman:** Conceptualization; data curation; formal analysis; investigation; methodology; project administration; resources; supervision; validation; visualization; writing – original draft; writing – review and editing. **Nicholas Williams:** Conceptualization; data curation; formal analysis; investigation; methodology; project administration; supervision; validation; writing – original draft; writing – review and editing. **Therese Dela Rueda:** Conceptualization; formal analysis; investigation; methodology; project administration; writing – original draft; writing – review and editing. **Edward Xiao:** Investigation; methodology; supervision; writing – original draft; writing – review and editing. **Arina Caliman:** Methodology; supervision; validation; writing – original draft; writing – review and editing. **James L. Chen:** Conceptualization; methodology; project administration; supervision; writing – review and editing. **Patrick J. McGahan:** Conceptualization; project administration; supervision; validation; writing – review and editing.

## FUNDING INFORMATION

None.

## CONFLICT OF INTEREST STATEMENT

J.L.C. is an educational consultant for Arthrex and receives compensation for medical educational lectures and instruction only.

## CONSENT

Written informed consent was obtained from the patient to publish this report in accordance with the journal's patient consent policy.

## Supporting information


Video S1.

## Data Availability

Data available on request from the authors.
